# Supplementary material for: Shortcut to synchronization in classical and quantum systems
Source: Sci Rep. 2023 Jan 9;13:453. doi: 10.1038/s41598-022-27130-w (PMC9829672; doi:10.1038/s41598-022-27130-w)
Supplement: Supplementary file 1 — Supplementary Information. [file 41598_2022_27130_MOESM1_ESM.pdf]

Suppplementary Information for

Shortcut to synchronization in classical and quantum systems

François Impens[1,+], David Guéry-Odelin[2,*]

[1]Instituto de Física, Universidade Federal do Rio de Janeiro, Rio de Janeiro, RJ 21941-972, Brazil
[2] Paul Sabatier University, Toulouse III, FeRMI, CNRS, France

Corresponding authors: [+]impens@if.ufrj.br, [*]dgo@irsamc.ups-tlse.fr

This PDF file inlcludes:

- Additional details on the obtention of shortcut-to-synchronization in classical and quantum van der Pol oscillators.
- Equation numbers refer to equations of the main article.

Shortcut-to-synchronization in a classical van der Pol oscillator

We detail here the procedure to design of a shortcut-to-synchronization from the initial point $(x_0, y_0) = (0,0)$. We first solve the trajectory of a sinusoidally driven van-der-Pol oscillator with $\varepsilon_0 = 1.5/T_0$ and obtain the branching point $(x_\infty, y_\infty) \simeq (0.29, 1.05)$ corresponding to $t_\infty = 50.125 \times T_0$. The proximity of this branching point to the "vertical" of the initial position (x_0, y_0) enables a fast shortcut with the amplitude $\varepsilon(t)$.

We first define a system trajectory of the form $y_{\text{short},\gamma}(t) = P_\gamma(t/\tau)$ with the polynomial $P_\gamma(u) = y_\infty + y_0'(t_\infty)\tau(u-1) + (y_0 - y_\infty + y_0'(t_\infty)\tau)(u-1)^2 + \gamma u(u-1)^2$. The chosen trajectory fulfills, for any value of the parameter γ, the required boundary conditions $y_{\text{short},\gamma}(0) = y_0$ and $y_{\text{short},\gamma}(\tau) = y_\infty$ associated respectively to the initial and final shortcut times. The additional condition $y'_{\text{short},\gamma}(\tau) = y_0'(t_\infty)$ provides a continuity of the driving amplitude between the transient and sinusoidal part.

To determine the correct shortcut trajectory and fix the γ parameter, we use a self-consistency argument: by virtue of Eq.(1) of the main text, when the system goes along the trajectory $y_{\text{short},\gamma}(t)$, the coordinate motion $x(t)$ follows a differential equation where $y_{\text{short},\gamma}(t)$ acts as a driving term:

$$\dot{x} = \omega_0 y_{\text{short},\gamma} + \kappa_1 x - 2\kappa_2(x^2 + y^2_{\text{short},\gamma})x.$$

With the considered initial condition $x(0) = x_0 = 0$, each value of γ yields a corresponding solution $x_{\text{short},\gamma}(t)$ and final coordinate $x_{\text{short},\gamma}(\tau)$ at the time τ. For the "magic"value γ_0, the final coordinate reaches the target, i.e. $x_{\text{short},\gamma_0}(\tau) = x_\infty$. Then, the trajectory $(x_{\text{short},\gamma_0}(t), y_{\text{short},\gamma_0}(t))$ reaches the branching point (x_∞, y_∞) at $t = \tau$, and can thus be choses as shortcut trajectory. For the parameters above, one finds numerically $\gamma_0 \simeq -9.3532$. The corresponding driving amplitude $\varepsilon_{\text{short}}(t)$ is derived from Eq. (1) of the main text as

$$\varepsilon_{\text{short}}(t) = -2[\dot{y}_{\text{short},\gamma_0}(t) + \omega_0 x_{\text{short},\gamma_0}(t) - \kappa_1 y_{\text{short},\gamma_0}(t) + 2\kappa_2(x^2_{\text{short},\gamma_0}(t) + y^2_{\text{short},\gamma_0}(t))y_{\text{short},\gamma_0}(t)]$$

for $t \leq \tau$. For $t > \tau$, the sinusoidal driving is resumed $\varepsilon(t) = \varepsilon_0 \cos(\omega t + \varphi)$. The phase φ is fixed as follows. At time τ, the system is at a position that would be reached under a plain sinusoidal driving $\varepsilon_0(t)$ at time t_∞. For our strategy to be sucessful, the system must be subject to a driving $\varepsilon(t)$ such that $\varepsilon(t-\tau) = \varepsilon_0(t - t_\infty)$ for $t > \tau$. A suitable choice is thus $\varphi = \omega(t_\infty - \tau)$.

Shortcut-to-synchronization in a quantum van der Pol oscillator

We detail the procedure for the shortcuts considered in Figs. 2 and 3 in the weakly/strongly non-linear regimes. We solve Eq. (2) of the main text in a quantum subspace corresponding to the N lowest-energy level of the harmonic oscillator. It is sufficient to consider $N = 40$, as higher-energy quantum states are irrelevant for the considered initial states and Hamiltonians.

In the weakly nonlinear regime, for a sinusoidal driving with $\varepsilon_1 = 1$, $\varepsilon_2 = 0$, one finds the stationary mean position $\alpha_\infty = x_\infty + iy_\infty \simeq -0.86 - 0.38i$ and the corresponding middle-point $\alpha_m = \frac{1}{2}(\alpha_0 + \alpha_\infty)$. For a generic intermediate point $\alpha_I = \alpha_m + i\Delta y$, we use a piece-wise defined path $\langle\alpha\rangle_t^{(1)} = \alpha_0 + 2(\alpha_I - \alpha_0)t/\tau$ for $0 \leq t \leq \tau/2$ and $\langle\alpha\rangle_t^{(1)} = \alpha_I + 2(\alpha_\infty - \alpha_I)t/\tau$ for $\tau/2 \leq t \leq \tau$. The driving amplitudes $\varepsilon_{1,2}^{(1)}(t)$ can be expressed from Eq. (4) of the main text with the semiclassical approximation:

$$\frac{1}{2}\left(\varepsilon_2^{(1)}(t) + i\varepsilon_1^{(1)}(t)\right) = -\frac{d\langle\alpha\rangle_t^{(1)}}{dt} - i\Delta\langle\alpha\rangle_t^{(1)} + (\kappa_1 + 2\kappa_2)\langle\alpha\rangle_t^{(1)} - 2\kappa_2|\langle\alpha\rangle_t^{(1)}|^2\langle\alpha\rangle_t^{(1)}$$

As an example, we consider the weakly nonlinear regime with a shortcut duration $\tau = 2$ and $\Delta y = 0$. The quantum trajectory on the time interval $0 \leq t \leq \tau/2$ starts at the initial point $\alpha_0 = -1 + i$ and ends at the intermediate point $\alpha_m \simeq -0.93 + 0.34i$. A numerical resolution of Eq.(2) with the amplitudes $\varepsilon_{1,2}^{(1)}(t)$ yields a first offset $\Delta\alpha^{(1)} = \langle x\rangle_{\tau/2} - x_m + i(\langle y\rangle_{\tau/2} - y_m)$. As indicated in the main text, we iterate the procedure with corrected trajectories $\langle\alpha\rangle_t^{(n)} = \langle\alpha\rangle_t^{(n-1)} - \Delta\alpha^{(n-1)}$. From successive iterations, one obtains $\Delta\alpha^{(1)} \simeq 0.34 - 0.27i$, $\Delta\alpha^{(2)} \simeq 0.038 - 0.041i$ and $\Delta\alpha^{(3)} \simeq (2.2 - 5.2i) \times 10^{-3}$. For the strongly nonlinear regime with a shortcut of duration $\tau = 0.5$ and $\Delta y = 0$, considering the intermediate point $\alpha_m \simeq -0.63 + 0.31i$ and time interval $0 \leq t \leq \tau/2$, our procedure delivers the successive offsets $\Delta\alpha^{(1)} \simeq 0.23 - 0.18i$, $\Delta\alpha^{(2)} \simeq (-8.6 + 8.0i) \times 10^{-3}$, $\Delta\alpha^{(3)} \simeq (6.1 - 3.4i) \times 10^{-4}$. The convergence is fast and a few iterations are sufficient for the purpose of driving the mean position close to its target. In our example, after three iterations the error on the mean position becomes irrelevant: the speed of quantum synchronization is then mostly limited by a mismatch in the third (and higher-order) moments with respect to their stationary values. The convergence of the iterative process increases when shorter time intervals are considered between the initial and intermediate points. For a given duration, the convergence is faster in the weakly nonlinear regime - the semi-classical approximation is more accurate in this case.
